# Surface structure influences contact killing of bacteria by copper

**DOI:** 10.1002/mbo3.170

**Published:** 2014-04-17

**Authors:** Marco Zeiger, Marc Solioz, Hervais Edongué, Eduard Arzt, Andreas S Schneider

**Affiliations:** 1 INM - Leibniz Institute for New MaterialsCampus D2 2, 66123, Saarbrücken, Germany; 2 Department of Plant Physiology and Biotechnology, Tomsk State UniversityTomsk, Russia; 3 University Yaounde IBP 812, Yaounde, Cameroon; 4 Saarland UniversityCampus D2 2, 66123, Saarbrücken, Germany

**Keywords:** Antibacterial activity, contact killing, copper surfaces, electrodeposition, nosocomial infection

## Abstract

Copper kills bacteria rapidly by a mechanism that is not yet fully resolved. The antibacterial property of copper has raised interest in its use in hospitals, in place of plastic or stainless steel. On the latter surfaces, bacteria can survive for days or even weeks. Copper surfaces could thus provide a powerful accessory measure to curb nosocomial infections. We here investigated the effect of the copper surface structure on the efficiency of contact killing of *Escherichia coli*, an aspect which so far has received very little attention. It was shown that electroplated copper surfaces killed bacteria more rapidly than either polished copper or native rolled copper. The release of ionic copper was also more rapid from electroplated copper compared to the other materials. Scanning electron microscopy revealed that the bacteria nudged into the grooves between the copper grains of deposited copper. The findings suggest that, in terms of contact killing, more efficient copper surfaces can be engineered.

## Introduction

Metallic copper surfaces have been shown to rapidly kill a range of microorganisms and viruses (for a recent review, see Grass et al. 2011). This so called ‘contact killing’ has raised renewed interest in the use of copper for touch surfaces. In health care settings, copper holds great promise as an added measure to curb nosocomial infections and a number of hospital trials have been conducted or are underway. First studies showed that copper surfaces can diminish the bacterial surface-loads up to 90% as compared to surfaces of other materials (Casey et al. 2010; Marais et al. 2010; Mikolay et al. 2010; Rai et al. 2012; Schmidt et al. 2013) and can significantly reduce nosocomial infections (Salgado et al. 2013).

These findings have also raised an interest in understanding the mechanism of contact killing. Although this mechanism is still not fully understood, recent studies suggest that copper surfaces kills bacteria by a three-pronged attack: damage of the bacterial membrane, DNA degradation, and extensive intracellular damage (Espirito Santo et al. 2008, 2011; Weaver et al. 2010; Warnes and Keevil 2011; Warnes et al. 2011). The sequence of these events is still under debate and may in fact be different, depending on the microorganism (Warnes et al. 2012). However, a key event in the killing process appears to be the release of copper ions from the copper surface; these ions can in turn, not only lead to the generation of highly toxic hydroxyl radicals in a Fenton-type reaction but also inactivate metalloproteins by replacing the respective metal with copper. In *Escherichia coli*, it was shown that FeS clusters are specific targets of copper toxicity (Macomber and Imlay 2009). That released copper ions are important in contact killing is further supported by the finding that bacteria resistant to copper are killed more slowly, and by the retardation of contact killing by corrosion inhibitors (Elguindi et al. 2009, 2011; Zhu et al. 2012).

The importance of copper ions in contact killing implies that the nature of the metallic copper surface plays an important role. We here used microstructured copper surfaces to show that increasing the copper surface leads to more rapid copper release and concomitantly, more rapid contact killing of *E. coli*. Such copper surfaces could thus provide superior antimicrobial surfaces for use in health care settings.

## Materials and Methods

### Generation of copper coupons

Rolled copper foils (99.9% copper, Circuit Foil Luxembourg, Luxembourg) were used as starting material. Electrodeposition was performed using a three-electrode cell with an Ag/AgCl reference electrode and two copper foils of 2.5 × 2.5 × 0.05 cm at a distance of 2 cm from each other, functioning as working and counter electrode. Thus, one foil was used as target material to structure the surface, whereas the other foil was used as substrate. For copper deposition, a current density of 20 mA cm^−2^ was applied for 300 seconds in 40 mL of an electrolyte solution composed of 2.5 mol L^−1^ CuSO_4_ and 1.2 mol L^−1^ H_2_SO_4_. For the generation of polished copper surfaces, rolled copper was polished successively with 6 *μ*m, 3 *μ*m, and 1 *μ*m diamond paste for 5 min each. Afterward the copper surface was extensively cleaned with ethanol and distilled water.

### Copper ion release by copper surfaces

Copper ions released into the aqueous phase by coupons were measured by atomic absorption spectroscopy (AAS). To remove possible oxide layers, coupons were cleaned by successively dipping them for 5 sec each into 10% H_2_SO_4_, water, and 3% NaOH, followed by extensive washing in water. For copper release measurements, 25 *μ*L of 0.9% NaCl were applied to coupons, covering a surface area of 16.0 ± 2.1 mm^2^. After different incubation times at 22°C in a water-saturated atmosphere, a quantity of 20 *μ*L of liquid was removed and dissolved copper was analyzed by AAS with a Vario 6-MPE 50 instrument (Analytics Jena, Jena, Germany). Measurements were conducted in 20 min intervals over the course of 1 h and the average release rates determined by linear regression analysis.

### Measurement of contact killing

The antibacterial activity of the different copper coupons was tested according to the common wet plating method (Wilks et al. 2005; Noyce et al. 2006; Weaver et al. 2008; Molteni et al. 2010). Briefly, test coupons of 2.5 × 2.5 cm were disinfected by dipping in 76% ethanol and drying in air, followed by treatment as for AAS described above. The Gram-negative test organism, *E. coli* K12 (ATCC 23716), was grown aerobically overnight in Luria-Bertani (LB) broth at 37°C (Ausubel et al. 1995). A culture volume of 1 mL was mixed with 10 mL of 0.9% NaCl. Culture aliquots of 25 *μ*L, corresponding to ∼2 × 10^8^ colony forming units (cfu), were deposited on the test coupons and incubated at 37°C in water-saturated air. After different contact times, 20 *μ*L of the droplets were removed and diluted in 180 *μ*L of media consisting of 3.6 g L^−1^ KH_2_PO_4_, 7.2 g L^−1^ Na_2_HPO_4_ · 2H_2_O, 4.3 g L^−1^ NaCl, 1 g L^−1^ meat peptone and 0.1% Tween 80. Serial dilutions were applied to LB agar plates and following incubation for 24 h at 37°C, cfu were assessed. Plating bacteria in parallel in an identical fashion on polycarbonate was used as a control in all experiments.

### Electron microscopy

The surface topography of the copper coupons was analyzed by scanning electron microscopy with a FEI Quanta 400 instrument (FEI, Hillsboro, OR) operating in standard mode. To visualize *E. coli* in contact with the surfaces, 2 × 10^8^ cells in 25 *μ*L of 0.9% NaCl were applied to the surface and dried. Visualization was accomplished with a FEI Quanta 400 instrument operating in environmental scanning electron microscopy (ESEM) mode at 5 kV. Multiple images were acquired in all cases.

## Results and Discussion

### Surface structures

**Figure 1:**
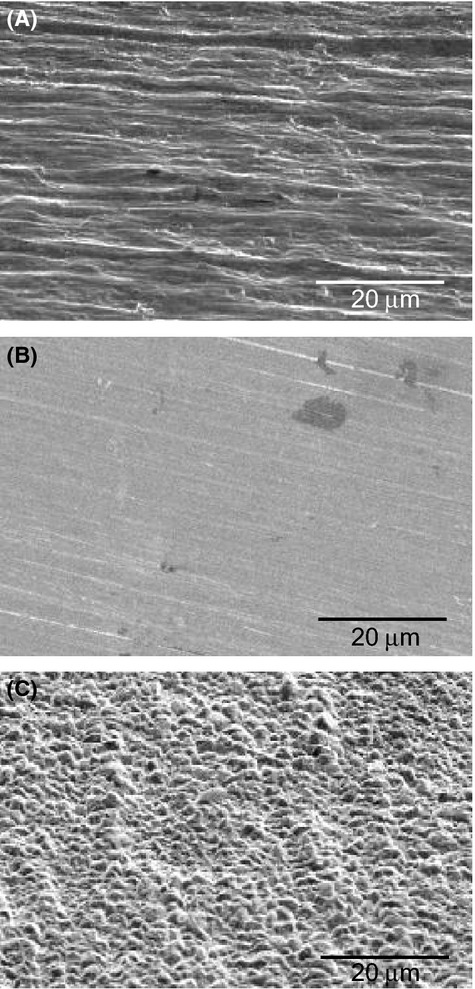
Scanning electron micrographs of copper surfaces. (A) Surface of industrial rolled copper foil, with the direction of milling being horizontal. (B) Surface of polished copper, with the direction of polishing from right to left, +20° off the normal. (C) Surface of electrodeposited copper.

To investigate the influence of the surface structure of metallic copper on contact killing, copper coupons with different surface topographies were produced. Using standard industrial rolled copper foil as starting material, rough copper surfaces were generated by electrodeposition and smooth copper surfaces by polishing. The surface topography of the copper coupons was visualized by scanning electron microscopy with a FEI Quanta 400 instrument (Fig. 1). The starting material, rolled copper, exhibited parallel groves with an average depth of 1–2 *μ*m, typical for this type of material. Polished copper on the other hand had a smooth surface structure, with comparatively minor grooves in the polishing direction. The dark patches in Figure 1B are irregularities of unknown origin which commonly occur in the copper starting material. Electroplating of copper finally resulted in a rough surface covered with copper grains, ranging in diameter from 1 to 5 *μ*m (Fig. 1C). Although electrodeposited and rolled copper surfaces displayed surface structures of similar size, they were qualitatively different. While electrodeposited copper displayed a pebbled surface structure, that of rolled copper featured uneven, parallel grooves.

### Copper release

**Figure 2:**
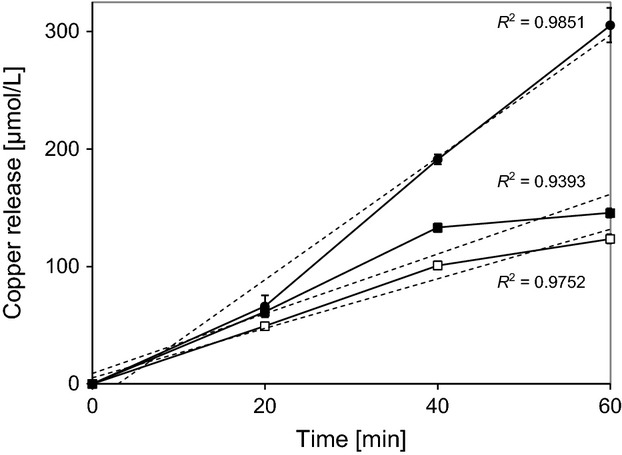
Measurement of copper release by different copper surfaces. Copper was measured as described under Materials and Methods. ▪, rolled copper; •, electrodeposited copper; □, polished copper. Measurements were carried out in triplicates and the error bars indicate standard deviations. The dashed lines show the linear regressions and the corresponding *R*^2^ values are given in the Figure.

It has previously been shown that there is a correlation between the release of ionic copper by the metal surface and the rate of contact killing. Copper ion release by the different copper surfaces was thus investigated. As shown in Figure 2, copper release by the three copper surfaces was essentially linear for 40 min, but decreased slightly thereafter for deposited and polished copper. Initial copper release rates were 0.42 nmol min^−1^ cm^−2^ for rolled copper, 0.33 nmol min^−1^ cm^−2^ for polished copper, and 0.77 nmol min^−1^ cm^−2^ for deposited copper, based on a contact area of 16.0 ± 2.1 mm^2^. These release rates raised the copper concentration in the aqueous phase to 0.16, 0.13 and 0.32 mmol/L in an hour. Surprisingly, copper release by rolled copper was only 20% higher than by polished copper, in spite of the much rougher surface aspect of this material. We have currently no explanation for this phenomenon. Copper release by deposited copper was, however, twofold higher than by the aforementioned copper surfaces. Electrodeposition leads to a fine-grained surface structure which is apparently more reactive toward an aqueous phase than either rolled or polished copper.

### Contact killing

**Figure 3:**
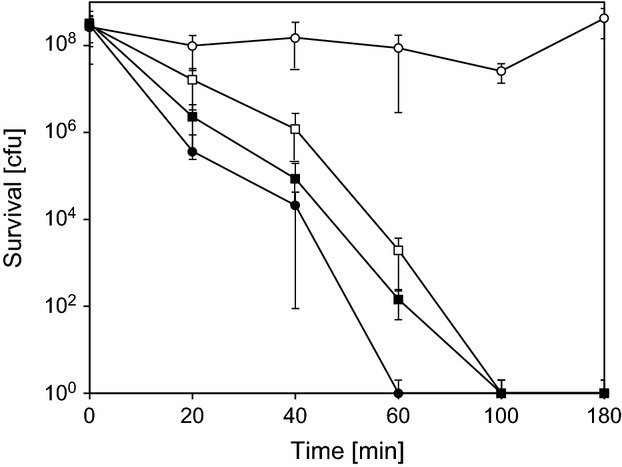
Survival of *E. coli* K12 on different copper surfaces. Approximately 2 × 10^8^ bacteria were applied to different copper surfaces and survival was assessed after different times of incubation as described under Materials and Methods. ○, Polycarbonate; ▪, rolled copper; •, electrodeposited copper; □, polished copper. Measurements were carried out in triplicates and the error bars indicate standard deviations.

When bacterial suspensions were applied to polycarbonate control coupons, no significant killing could be observed over 180 min (Fig. 3). On coupons of rolled or polished copper, 2 × 10^8^ bacteria were completely killed in 100 min. On electrodeposited copper, on the other hand, complete killing only took 60 min. The higher rate of copper release by electrodeposited copper thus correlated with its stronger antimicrobial properties, suggesting that the rate of copper release is a key factor in contact killing. In a previous study, it was similarly shown that the antibacterial activity of rough, cold sprayed copper surfaces was more pronounced than that of smooth, plasma sprayed or wire arc sprayed copper surfaces (Champagne and Helfritch 2013). Unfortunately, copper release by these different copper surfaces was not assessed and no direct comparison to standard copper surfaces was made. But is appears clear that the surface structure of copper has an influence on the efficiency of contact killing and a major factor for this may be the differing release of ionic copper.

### Visualization of bacteria on copper surfaces

**Figure 4:**
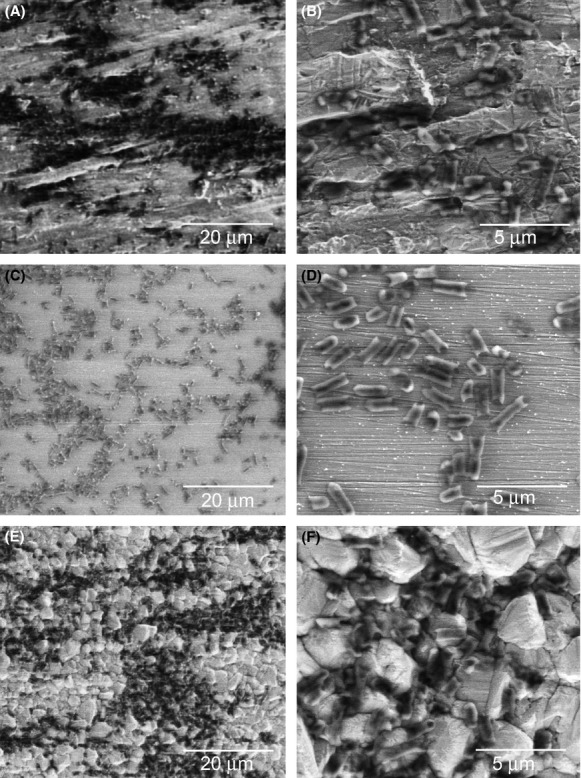
Scanning electron micrographs of *E. coli* on copper surfaces. Approximately 2 × 10^8^ cells were applied to the copper surfaces and visualized as described under Materials and Methods. (A, B) Rolled copper, (C, D) polished copper, (E, F) electrodeposited copper.

To obtain information about the behavior of bacterial cells on the different copper surfaces, we visualized them by scanning electron microscopy without using a staining procedure. The surface aspects in these pictures look somewhat different from those in Figure 1, due to the different imaging technique which had to be used (see Materials and Methods). On rolled copper, the spread of cells on the surface was uneven, but it did not appear to follow the uneven surface structure (Fig. 4A and B). In contrast, cells spread much more evenly on polished copper (Fig. 4C and D). On deposited copper, cell spread was comparable to that on rolled copper, but the cells nudged into the grooves between the copper grains (Fig. 4E and F). It has recently been shown that bacteria-copper contact is important in the contact killing process (Mathews et al. 2013). The lodging of bacteria into the grooves of deposited copper would of course enhance bacteria-metal contact, which could be an additional reason for the enhanced contact killing by deposited copper. Further work will have to address, whether enhanced copper release or more extensive surface contact is the primary reason for the increased efficiency of deposited copper in contact killing.

The antimicrobial properties of copper and copper alloys make them materials of choice for the fabrication of antimicrobial surfaces in health care settings or food processing industries. The finding reported here that the antimicrobial activity of electrodeposited copper is superior to that of rolled or polished copper may be an important engineering consideration in this connection. Electrodeposition of copper is a straight-forward, inexpensive industrial process which can be applied to a variety of metals. This could be a cost-effective method to make surfaces antimicrobial.
